# Structural Characterization of Human Heat Shock Protein 90 N-Terminal Domain and Its Variants K112R and K112A in Complex with a Potent 1,2,3-Triazole-Based Inhibitor

**DOI:** 10.3390/ijms23169458

**Published:** 2022-08-21

**Authors:** Giusy Tassone, Marco Mazzorana, Stefano Mangani, Elena Petricci, Elena Cini, Giuseppe Giannini, Cecilia Pozzi, Samuele Maramai

**Affiliations:** 1 Department of Biotechnology, Chemistry and Pharmacy, Department of Excellence 2018–2022, University of Siena, Via Aldo Moro 2, I-53100 Siena, Italy; 2 Diamond Light Source Ltd., Diamond House, Harwell Science & Innovation Campus, Didcot, Oxfordshire OX11 0DE, UK; 3 R&D Alfasigma S.p.A., I-00071 Pomezia, Italy

**Keywords:** Hsp90, X-ray crystallography, protein-inhibitor complex, 1,2,3-triazole-based inhibitor, selectivity

## Abstract

Heat shock protein 90 (Hsp90) is a ubiquitous molecular chaperone that stabilizes client proteins in a folded and functional state. It is composed of two identical and symmetrical subunits and each monomer consists of three domains, the N-terminal (NTD), the middle (MD), and the C-terminal domain (CTD). Since the chaperone activity requires ATP hydrolysis, molecules able to occupy the ATP-binding pocket in the NTD act as Hsp90 inhibitors, leading to client protein degradation and cell death. Therefore, human Hsp90 represents a validated target for developing new anticancer drugs. Since protozoan parasites use their Hsp90 to trigger important transitions between different stages of their life cycle, this protein also represents a profitable target in anti-parasite drug discovery. Nevertheless, the development of molecules able to selectively target the ATP-binding site of protozoan Hsp90 is challenging due to the high homology with the human Hsp90 NTD (hHsp90-NTD). In a previous work, a series of potent Hsp90 inhibitors based on a 1,4,5-trisubstituted 1,2,3-triazole scaffold was developed. The most promising inhibitor of the series, JMC31, showed potent Hsp90 binding and antiproliferative activity in NCI-H460 cells in the low-nanomolar range. In this work, we present the structural characterization of hHsp90-NTD in complex with JMC31 through X-ray crystallography. In addition, to elucidate the role of residue 112 on the ligand binding and its exploitability for the development of selective inhibitors, we investigated the crystal structures of hHsp90-NTD variants (K112R and K112A) in complex with JMC31.

## 1. Introduction

Heat shock protein 90 (Hsp90) is the most abundant ATP-dependent molecular chaperone that plays a key role in the folding, stabilization, and function of a wide variety of client proteins within the cell [1]. It represents about 1–2% of the whole cytosolic proteomic load, although under stress conditions it can increase up to 4–6% [2]. This chaperone takes part in various cellular pathways, including signal transduction, protein degradation, and stress resistance [3,4]. Client proteins of Hsp90 include transcription factors, kinases, receptors, and other non-structurally related proteins. Hsp90 uses ATP hydrolysis to provide the energy inducing the proper folding of client proteins and preventing their aggregation [5]. Hsp90 is a functional homodimer composed of two identical subunits, each one consisting of three domains: the N-terminal (NTD), for ATP- and drug-binding and a co-chaperone interacting motif, the middle (MD), for client and co-chaperone binding, and the C-terminal (CTD) domain, for constitutive dimerization as well as co-chaperone and client interaction. The ATP-binding site is located centrally in the Hps90-NTD. Indeed, this domain is structured as a two-layer sandwich, formed by a β-sheet on one side and a cluster of α-helices on the other, delimiting the ATP-binding pocket, a flat-bottomed cone of about 15 Å in depth [6].

**Figure 1:**
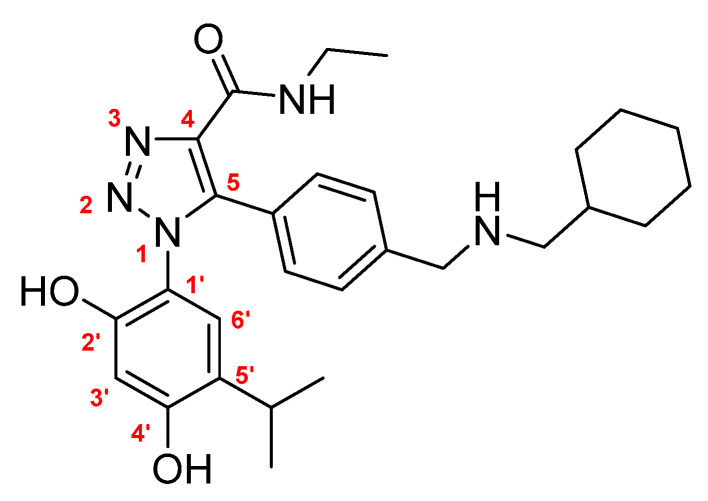
Chemical structure of JMC31. The atom numbering mentioned in the text is indicated.

Since the high flexibility of Hsp90 makes the structural characterization of the full-length protein challenging, the protein has been widely studied as isolated domains. Among them, the NTD, containing the ATP-binding site, represents the most studied and exploited domain, especially for the development of Hsp90 inhibitors. In fact, molecules targeting the active site behave as Hsp90 inhibitors by competing with ATP and preventing its hydrolysis. Abolishing chaperone activity leads to protein degradation and subsequent cell death. Since various oncoproteins and proteins linked to tumor progression and invasiveness are included among human Hsp90 clients, this chaperone is a validated drug target for the development of anticancer therapies [7,8,9,10]. On the other hand, Hsp90 plays a key role in the survival and proliferation of a wide variety of human protozoan parasites, making it a promising target for the treatment of various neglected diseases [9,11,12,13]. Indeed, the environmental differences encountered by the parasites moving from the insect vector to the mammalian host (including variations in temperature, pH, and ionic strength) stimulate the production of this protein [14]. In this way, Hsp90 allows the parasite to resist thermal stress and participates in the stabilization and enhancement of different client proteins essential for constitutive cell signaling and an adaptive response to the new environment. [15,16,17,18]. Nevertheless, the development of new anti-parasite drugs targeting Hsp90 is a daunting task, mainly due to the high conservation of the ATP-binding site between human and parasite proteins drastically limiting the identification of selective molecules [14,19]. In our former research work, we evaluated the role of nucleotide binding of Arg97 in *Leishmania braziliensis* Hsp90-NTD (a residue also conserved in other parasites) and of Lys112 in the human counterpart, to find new determinants to selectively target the parasite protein. These amino acids represent the only non-conserved residues in the ATP-binding pocket of human and parasite Hsp90-NTDs [20]. For this reason, they have been proposed as exploitable for the development of selective inhibitors [12]. Two variants of human Hsp90-NTD, K112R (representing the “*leishmanized*” protein) and K112A, have been generated and probed for nucleotide binding. To further investigate the contribution of these residues to the binding and selectivity of different ligands, we decided to analyze the crystal structures of hHsp90-NTD and its variants in complex with a previously reported inhibitor, here referred to as JMC31 (Figure 1), which is not structurally related to ATP [21,22]. JMC31 belongs to a new family of 1,4,5-trisubstituted triazole carboxamides that display high affinity toward hHsp90 associated with cell proliferation inhibition, both in the nanomolar range. Here, the 1,5-arrangement of the resorcinol, the aryl moieties, and the presence of a secondary amide in position 4 of the triazole ring were essential to achieve high activity.

This structural characterization allows us to discuss the contribution of residue 112 to the binding of a non-substrate-related ligand, and its exploitability for the synthesis of selective parasite Hsp90 inhibitors. To obtain additional insight in this field, we characterized the complex of JMC31 with the K112R variant, the “*leishmanized*” protein, and the K112A variant, specifically selected to remove the contribution of Lys112 to the ligand binding.

## 2. Results

### 2.1. Synthesis and Characterization of JMC31

*5-(4-(((Cyclohexylmethyl)amino)methyl)phenyl)-1-(2,4-dihydroxy-5-isopropylphenyl)-N-ethyl-1H-1,2,3-triazole-4-carboxamide (JMC31)*. The synthesis of the title compound was accomplished following a previously published procedure [21]. ESI-MS m/z: 492 [M + H]^+^, 514 [M + Na]^+^ (100); ^1^H NMR (400 MHz, MeOD) *δ* 7.30 (dd, *J* = 18.8, 7.4 Hz, 4H), 6.84 (s, 1H), 6.29 (s, 1H), 3.74 (s, 2H), 3.37 (q, *J* = 6.7 Hz, 2H), 3.16–3.02 (m, 1H), 2.39 (d, *J* = 6.1 Hz, 2H), 1.77–1.41 (m, 6H), 1.30–1.12 (m, 6H), 1.06 (d, *J* = 6.2 Hz, 6H), 0.87 (dd, *J* = 18.7, 7.5 Hz, 2H); ^13^C NMR (101 MHz, MeOD) *δ* 161.5, 157.1, 151.5, 140.5, 139.4, 137.9, 130.0, 127.7, 126.6, 125.6, 125.4, 114.8, 102.4, 54.8, 52.5, 36.9, 33.7, 30.9, 26.1, 25.9., 25.6, 21.6, 13.6. The purity of JMC31 was >95% by HPLC analysis.

### 2.2. Crystal Structures of hHsp90-NTD and Variants K122R and K112A in Complex with JMC31

The structures of hHsp90-NTD and its variants K112R and K112A in complex with JMC31 were determined to a resolution ranging from 2.10 to 2.32 Å (Table 1). In all complexes, the crystal asymmetric unit contains two independent molecules, having an overall structure highly conserved with those previously described for the native enzyme [20,23]. The protein chain was modeled completely apart from the first 14 and last 31 residues at the N and C terminus, respectively (the 20 residues composing the N-terminal His_6_-tag are also missing in the models).

**Figure 2:**
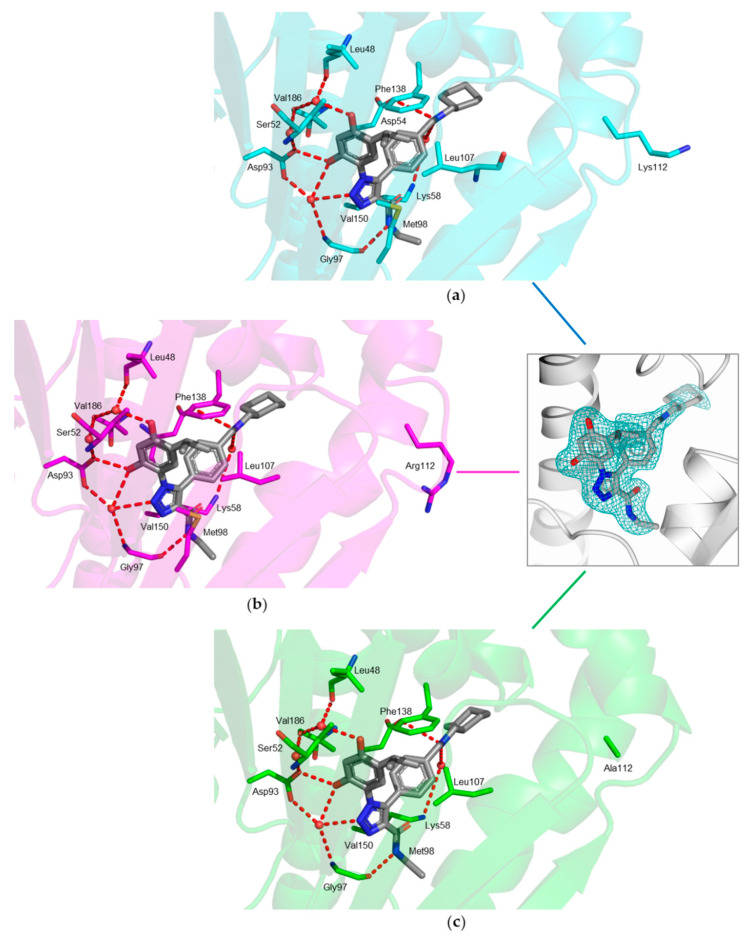
Active-site view of (**a**) hHsp90-NTD (cyan cartoon and carbons, residues in sticks) in complex with JMC31 (in sticks, grey carbons); (**b**) hHsp90-NTD K112R variant (magenta cartoon and carbons, residues in sticks) in complex with JMC31 (in sticks, gray carbons); (**c**) hHsp90-NTD K112A variant (green cartoon and carbons, residues in sticks) in complex with JMC31 (in sticks, gray carbons). The fitting of the JMC31 (in sticks, gray carbons) in the omit map (teal mash, contoured at the 3.0 σ level) is shown in the inset. In all figures, H-bonds are displayed as red dashed lines and water molecules as red spheres. Oxygen atoms are colored red, nitrogen blue, and sulfur yellow.

The ligand occupies the active pocket in both chains of all structures, and it shows a conserved binding mode stabilized by a tight network of interactions (Figure 2). The resorcinol hydroxyl moiety in position 2′ establishes both direct and water-mediated interactions with the carboxylate of Asp93. On the other hand, the resorcinol hydroxyl moiety in position 4′ is involved only in water-mediated interactions with Asp93 carboxylate, Leu48 carbonyl backbone, and Ser52 hydroxyl moiety. The isopropyl moiety occupies the pocket lined by Leu48, Leu107, Thr109, Phe138, Val150, and Val186. The triazole nitrogen in position 2 establishes a water-mediated interaction with the Gly97 backbone nitrogen. The nitrogen atom of the amide group forms an H-bond with the Gly97 backbone carbonyl. The nitrogen atom present in the aliphatic linker between the aryl and the terminal cyclohexane forms a direct H-bond with Asp54 and a water-mediated interaction with Lys58 (Figure 2). The terminal cyclohexane ring of JMC31 is exposed to the solvent. In all complexes, the residue in position 112, being lysine in hHsp90-NTD (Figure 2a), arginine in K112R (Figure 2b), and alanine in K112A (Figure 2c), points outside the catalytic cavity, thus is not involved in ligand binding within the active site. Our investigations are in line with what has already been observed for nucleotide binding, and confirmed that Lys112 in hHsp90-NTD, corresponding to Arg97 in protozoan parasites, does not intervene in the binding of this class of compounds.

## 3. Discussion

In our previous studies, we generated different variants of human Hsp90-NTD to deeply investigate the contribution of different amino acids to the binding of various nucleotides. The ATP-binding site in Hsp90-NTD is highly conserved across species, as seen between eukaryotic and protozoan parasite proteins [12,24,25]. The only exception is represented by one residue, namely Lys112 in hHsp90-NTD, which is replaced by a different amino acid in the protozoan counterpart (e.g., Arg97 in *Leishmania braziliensis* Hsp90-NTD [20]). Thus, this position has been already proposed as exploitable for the development of selective agents targeting only the parasite protein over the human counterpart [26,27]. Therefore, we decided to replace Lys112 with both an arginine (K112R), representing the parasite protein, and an alanine (K112A) [20]. Our structural investigations revealed that, even though both the parasite arginine and the human lysine are variably involved in the interaction with the nucleotides, their contribution is not crucial for the binding of substrate and related analogues. Indeed, abolishing the contribution provided by these residues does not alter the binding mode of the natural nucleotide substrate, as verified in our earlier studies by the mutation to alanine of this position (K112A variant). This confirms that the residue in position 112 does not substantially contribute to substrate binding between the different protein variants [20]. Its vicinity to the active site makes it worth exploring as an anchoring point for selective inhibitors reaching both the nucleotide binding pocket and the variable 112 position.

To evaluate the contribution of the same residues to the binding of non-substrate related ligands, we characterized the structure of hHsp90-NTD and its variants in complex with JMC31. This compound was previously reported as a potent inhibitor of hHsp90 and arose from a small library of compounds based on the 1,2,3-triazole scaffold [21]. In these molecules, the concomitant presence of a resorcinol-like moiety, an aryl group, and an alkyl amide in position 4 of the triazole ring represented essential features accounting for its potent inhibitory activity. JMC31 showed Hsp90 binding in the single digit nanomolar concentration in the fluorescence polarization (FP) assay. Furthermore, this compound displayed antiproliferative activity toward non-small cell lung carcinoma NCI-H460 with an IC_50_ of 2.1 nM, thus confirming Hsp90 as a validated target in cancer therapy [7].

In our structures, JMC31 is anchored to the ATP catalytic pocket by a tight network of conserved interactions. In particular, the resorcinol moiety of the ligand is involved in both direct and water-mediated H-bonds with Asp93 and Gly97 and further water-mediated interactions with Leu48 and Ser52 (Figure 2). These interactions are also responsible for substrate binding, as previously shown by the structural investigation of Hsp90s from various organisms [28]. In the hHsp90-NTD–JMC31 complex, the main moiety responsible for the binding in the ATP-catalytic cavity is resorcinol, a chemical motif shared with radicicol, a known Hsp90 inhibitor [28,29]. The resorcinol is entrapped within the active site, where the hydroxyl group in position 4 displaces one of the three conserved water molecules known to play a crucial role in substrate binding (Figure 2) [28]. As mentioned above, the phenolic hydroxyl groups, one nitrogen atom (N2) of the triazole ring and the amide of JMC31, establish a tight network of bonds with Asp93, Gly97, and water molecules, which are reported as key interactions for the inhibitor binding and activity [24,25]. These findings agree with the molecular modeling studies and computational analysis reported in the original research work involving JMC31 and its analogues. In fact, as suggested by the docking simulation, these compounds are accommodated in the active site and the binding mode observed in the crystal structure is in line with the predicted docking pose [21].

**Figure 3:**
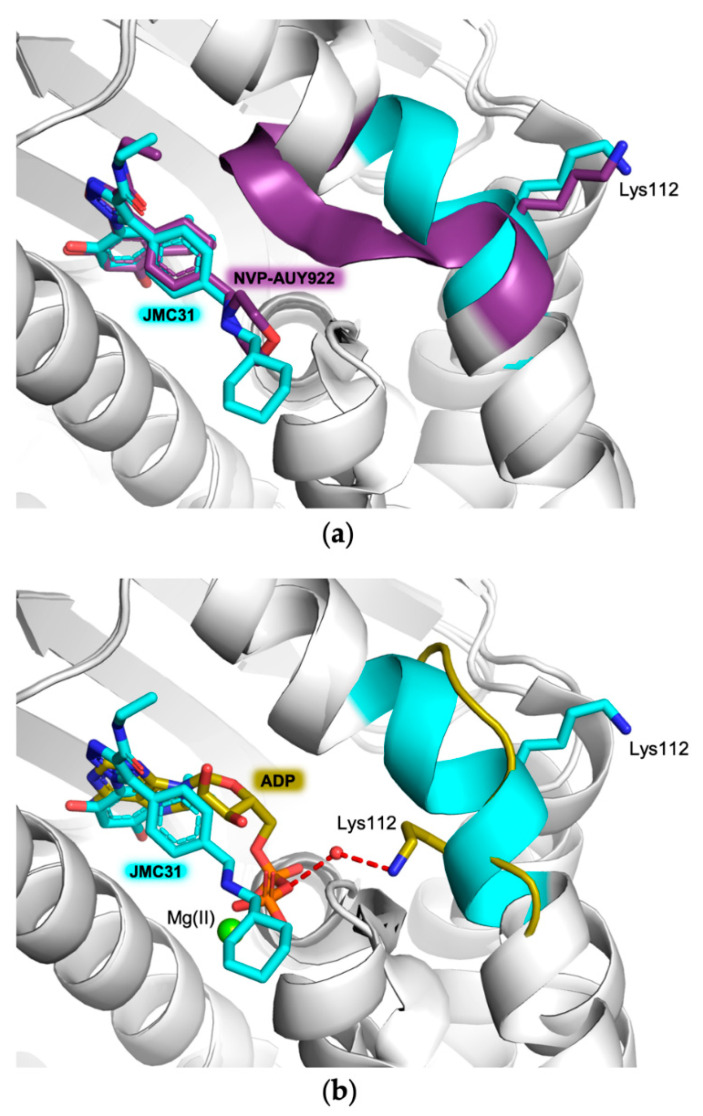
Structural comparison between the flexible regions of hHsp90-NTD (residues 106–113, cyan cartoon and carbons) in complex with (**a**) JMC31 (in stick, cyan carbons) and NVP-AUY922 (in stick, purple carbons, PDB code: 2VCI [32]; (**b**) JMC31 (in stick, cyan carbons) and ADP (in stick, olive carbons, PDB code: 6GP6 [20]. In both panels, for clarity, only Lys112 is highlighted in stick in the same color of the protein to which it belongs. Magnesium ion is displayed as a green sphere.

The peculiar network of interactions is conserved in each hHsp90-NTD variant (Figure 2) and observed both in the substrate and in other ligands and/or inhibitors [15,20,24,28]. Nevertheless, the region consisting of residues from 106 to 114 is highly flexible and can assume two main conformations. The state in which this region is proximal to the catalytic pocket is known as the “closed” conformation, whereas the one moved far from the active site is called “open”. The transition between these two states is needed for the substrate entering the active site, since in the closed conformation the region buries the ATP-binding pocket, preventing the formation of the complex [12,20,23]. However, intermediate conformations have also been observed in the structures of Hsp90-NTDs, supporting the functional role of this region and its influence on the potency of Hsp90 ligands [23,30,31]. The two extreme cases can be seen by comparing the structures of hHsp90-NTDs in complex with JMC31 and of a reported ligand structurally related to our compound, NVP-AUY922 (PDB code: 2VCI, [32] Figure 3a): in the presence of JMC31, the region has a longer α-helix with JMC31 than in the closed conformation associated with NVP-AUY922 binding. Despite these different arrangements, Lys112 points outside the active site when either JMC31 or other ligands are accommodated in the ATP-binding site. However, when ADP is bound (PDB code: 6GQ6, [20]), the region is in its open conformation and Lys112 points directly towards the catalytic cavity, engaging in a water-mediated interaction with the ADP α-phosphate (Figure 3b).

In agreement with our findings, the contribution of Lys112 to the ligand binding is therefore not crucial and both the human lysine and the parasite arginine are not involved in the interaction with JMC31 (Figure 2a,b). The binding mode of the ligand is not altered upon the removal of the contribution provided by these residues, as verified by the replacement with alanine in the K112A variant (Figure 2c).

## 4. Materials and Methods

### 4.1. Synthesis of JMC31

The synthetic procedures and analysis were reproduced from the previously reported synthesis of JMC31 [21]. Electrospray ionization mass spectrometry (ESI-MS) was performed by an Agilent 1100 series liquid chromatograph system with an API-ES interface. Mass spectra were acquired in positive mode scanning over the mass range *m/z* of 150–1500. ^1^H NMR and ^13^C NMR spectra were recorded on a Bruker Advance DPX400 operating at 400 MHz, using the residual signal of the deuterated solvent as the internal standard. The value of chemical shifts (*δ*) is given in ppm and coupling constants (*J*) are given in hertz (Hz). Splitting patterns are described as singlet (s), doublet (d), triplet (t), quartet (q), doublet of doublet (dd), and multiplet (m). The chemical purity of the final compound was determined using an Agilent 1260 Infinity instrument comprising a binary pump, an autosampler, a UV-DAD, and an ESI-MS detector. The chromatographic separation was performed with an Agilent Poroshell 120 EC-C18 column (4.6 mm × 100 mm, 2.7 μm), injecting 5 μL of the sample. Analyses were carried out with gradient elution, solvent A (0.1% formic acid in water), and solvent B (0.1% formic acid in acetonitrile), 90:10 to 10:90 over 6 min, at the flow rate of 2 mL/min, and a UV detector at 254 nm. The compound purity was ≥95.0%.

### 4.2. Proteins Expression, Purification, and Crystallization

Recombinant His_6_-tag hHsp90-NTD and its variants K112R and K112A were expressed in *E. coli* as His_6_-tag proteins and purified following established protocols [20]. Crystals of hHsp90-NTD K112A in complex with JMC31 were obtained by co-crystallization using the sitting drop vapor diffusion technique at 8 °C [33], according to the reported protocols [20,23]. Briefly, samples for the crystallization experiment were prepared by adding 5 mM JMC31 (dissolved in dimethyl sulfoxide, DMSO) to the proteins (20 mg mL^−1^, in 500 mM sodium chloride and 20mM Tris-Cl, pH 7.5) and incubating 1 h at 4 °C. Crystallization drops, consisting of equal volumes of these samples and precipitant solution, were equilibrated over a 500 μL reservoir at 8 °C. For hHsp90-NTD and its variant K112R, the precipitant formulation was 25% *wt*/*vol* PEG-4000, 200 mM magnesium chloride and 100 mM TRIS-HCl, pH 8.5, while for the K112A mutant, we used 25% *wt*/*vol* PEG-2000, 200 mM magnesium chloride and 100 mM sodium cacodylate, pH 6.5. Crystals of the complexes were cryoprotected by the addition of 10% *wt*/*vol* PEG-2000 to the precipitant solution of hHsp90-NTD K112A and 20% PEG-400 to that of hHsp90-NTD and its variant K112R, and flash frozen in liquid nitrogen.

### 4.3. Data Collection, Structure Solution and Refinement

Diffraction data were collected at 100 K using synchrotron radiation at the Diamond Light Source (DLS, Didcot, UK) beamline I04 equipped with the Eiger2 XE 16M detector. Data were integrated using XDS [34] and scaled with SCALA [35,36] from the CCP4 suite [37]. The crystals of the complexes belonged to the tetragonal space group P4_3_2_1_2 with two protein chains in the asymmetric unit. Structures were solved by the molecular replacement technique as implemented in the software MOLREP [38]. The structure of hHsp90-NTD in complex with ADP (PDB id 6GQ6 [20], excluding non-protein atoms and water molecules) was used as a searching model. All structures were refined by alternating manual correction and rebuilding using the molecular graphic software Coot [39] and cycles of automated refinement with REFMAC5 [40] from the CCP4 suite [37]. Water molecules were added through the program Coot [39] and checked both manually and automatically. In all complexes, the inspection of the Fourier difference map clearly showed the presence of JMC31, which was manually placed inside the catalytic cavity according to the electron density. The final models were inspected manually and checked with Coot and PROCHECK [41] and then validated through the Protein Data Bank (PDB) deposition tools. Structural figures were generated using the molecular graphic software CCP4 mg [42] and Pymol [43]. Data collection, processing, and refinement statistics are summarized in Table 1. Final coordinates and structure factors were deposited in the Protein Data Bank (PDB) under the codes 8AGI (hHsp90-NTD–JMC31), 8AGL (hHsp90-NTD K112A–JMC31), and 8AGJ (hHsp90-NTD K112R–JMC31).

## 5. Conclusions

Hsp90 is a molecular chaperone highly conserved from mammals to protozoan parasites. It is essential to promote the folding, stabilization, and assembly of nascent proteins. Due to its key role, Hsp90 represents a promising target for the development of new anticancer and anti-parasite drugs. The high conservation of the residues responsible for the recognition and catalysis of the substrate among the species makes the identification of selective inhibitors a challenging task. Starting from the previously developed compound JMC31, known to be a potent inhibitor of hHsp90, we investigated the structure of its complex with hHsp90-NTD. To further elucidate the contribution of residue 112 on the ligand binding, the only non-conserved residue between the catalytic pocket of human and parasite proteins, we also solved the crystal structures of our ligand in complex with K112R and K112A variants, structurally characterized in a previous work. These findings show that the scaffold of JMC31 may not be directly exploited to develop protozoan-selective inhibitors as it fails to force the protein in its fully open conformation, in which Lys112/Arg97 point towards the active site.

## Figures and Tables

**Table 1:** Data collection and refinement statistics. Values for the outer shell are given in parentheses.

	hHsp90-NTD JMC31	hHsp90-NTD K112R JMC31	hHsp90-NTD K112A JMC31
PDB ID codes	**8AGI**	**8AGL**	**8AGJ**
Data Collection Statistics			
Diffraction source	I04 (DLS)	I04 (DLS)	I04 (DLS)
Wavelength (Å)	0.9795	0.9795	0.9795
Temperature (K)	100	100	100
Detector	Eiger2 XE 16M	Eiger2 XE 16M	Eiger2 XE 16M
Crystal–detector distance (mm)	258.4	294.9	258.4
Exposure time per image (s)	0.2	0.2	0.2
Space group	P4_3_2_1_2	P4_3_2_1_2	P4_3_2_1_2
No. of subunits in ASU	2	2	2
*a = b*, *c* (Å)	72.90, 212.19	72.86, 210.53	73.03, 209.29
Resolution range (Å)	212.19–2.10 (2.21–2.10)	210.53–2.20 (2.32–2.20)	209.29–2.32 (2.45–2.32)
Total no. of reflections	397,371 (53116)	394,512 (57473)	333,207 (50672)
No. of unique reflections	30,642 (4410)	29,864 (4245)	25,323 (3654)
Completeness (%)	88.6 (89.7)	100.0 (100.0)	99.1 (100.0)
Redundancy	13.0 (12.0)	13.2 (13.5)	13.2 (13.9)
〈*I*/σ(*I*)〉	24.5 (2.8)	25.7 (2.7)	8.5 (2.5)
*R*_meas_	0.057 (0.788)	0.051 (0.973)	0.170 (1.356)
Overall *B* factor from Wilson plot (Å^2^)	40.8	55.2	42.9
Refinements Statistics			
Resolution range (Å)	68.94–2.10 (2.15–2.10)	68.86–2.20 (2.56–2.20)	68.95–2.32 (2.38–2.32)
Completeness (%)	88.7 (100.0)	100.0 (100.0)	98.6 (98.5)
No. of reflections, working set	29,011 (2383)	28,381 (2048)	23,842 (1727)
No. of reflections, test set	1547 (124)	1393 (91)	1289 (99)
Final *R*_cryst_	0.2099 (0.312)	0.2295 (0.350)	0.2219 (0.301)
Final *R*_free_	0.2764 (0.393)	0.2974 (0.374)	0.2841 (0.344)
No. of non-H atoms			
Protein	3202	3195	3178
JMC31	72	72	72
Water	161	125	156
Total	3435	3392	3406
R.m.s. deviations Bonds (Å)	0.009	0.007	0.009
Angles (°)	1.657	1.566	1.632
Average *B* factors (Å^2^)	45.4	61.5	51.7
Estimate error on coordinates based on R value (Å)	0.221	0.246	0.304
Ramachandran plot			
Most favored (%)	98.6%	94%	91.6%
Allowed (%)	1.4%	6%	8.4%
RSCC JMC31 chain A, B	0.91, 0.88	0.97, 0.89	0.95, 0.94

## Data Availability

Crystal structure final coordinates and structure factors are available in the PDB (www.rcsb.org (accessed on 20 July 2022)) under the codes 8AGI (hHsp90-NTD–JMC31), 8AGL (hHsp90-NTD K112R–JMC31), and 8AGJ (hHsp90-NTD K112A–JMC31).
